# Occurrence of comorbidities in newly diagnosed type 2 diabetes patients and their impact after 11 years’ follow-up

**DOI:** 10.1038/s41598-021-90379-0

**Published:** 2021-05-26

**Authors:** Sophia Eilat-Tsanani, Avital Margalit, Liran Nevet Golan

**Affiliations:** 1 The Department of Family Medicine, Emek Medical Center, Yitzhak Rabin Boulevard 21, 1834111 Afula, Israel; 2 Clalit Health Services, Northern Region, Nof Hagalil, Israel; 3 Azrieli Faculty of Medicine, Bar Ilan University, Safed, Israel; 4 Yoseftal Medical Center, Eilat, Israel

**Keywords:** Cancer, Cardiovascular diseases, Endocrine system and metabolic diseases

## Abstract

The burden of type 2 diabetes is growing, not only through increased incidence, but also through its comorbidities. Concordant comorbidities for type 2 diabetes, such as cardiovascular diseases, are considered expected outcomes of the disease or disease complications, while discordant comorbidities are not considered to be directly related to type 2 diabetes and are less extensively addressed under diabetes management. Here we show that the combination of concordant and discordant comorbidities appears frequently in persons with diabetes (75%). Persons with combined comorbidities visited family physicians more than persons with discordant, concordant or no comorbidity (17.3 ± 10.2, 11.6 ± 6.5, 8.7 ± 6.8, 6.3 ± 6.6 visits/person/year respectively, p < 0.0001). The risk of death during the study period was highest in persons with combined comorbidities and discordant only comorbidities (HR = 33.4; 95% CI 12.5–89.2 and HR = 33.5; 95% CI 11.7–95.8), emphasizing the contribution of discordant comorbidities to the outcome. Our study is unique as a long-term follow-up of an 11-year cohort of 9725 persons with new-onset type 2 diabetes. The findings highlight the contribution of discordant comorbidity to the burden of the disease. The high prevalence of the combination of both concordant and discordant comorbidities, and their appearance before the onset of type 2 diabetes, indicates a continuum of morbidity.

## Introduction

The prevalence of chronic diseases has increased dramatically in developed countries in recent decades^1^. Parallel to the increased prevalence of type 2 diabetes, the burden of the disease and its comorbidities has become a growing concern for health providers^2^. The clustering of chronic diseases, known as multimorbidity, is common in type 2 diabetes^3,4^, and contributes substantially to the burden of the disease^5^. Advanced age, female gender and low socioeconomic status (SES) have been found to be associated with higher rates of multimorbidity^3,4,6^.

Some diseases, such as hypertension, ischemic heart disease, nephropathy and retinopathy, are considered concordant to diabetes, since they represent parts of the same pathophysiologic risk profile and are considered expected outcomes of the disease or disease complications. Other diseases, such as depression, rheumatologic diseases, chronic lung disease and malignant diseases^6,7^, are considered "discordant" to diabetes, as they are not directly related to the pathogenesis of diabetes and do not share similar risk factors. The risks of both concordant and discordant comorbidities increase with time in persons with type 2 diabetes^6^. Concordant comorbidities, such as cardiovascular diseases, are treated as part of the detailed protocol of care for diabetes management and are more likely to be the focus of the disease management plan^7^. In contrast, discordant comorbidities are less extensively addressed under diabetes management^7^.

Piette and Kerry elaborated on the substantial contribution of discordant comorbidities to health outcomes^8^. A number of subsequent studies reported that concordant diseases are more easily managed than discordant diseases, with better outcomes^9,10^. Managing multimorbidity in routine clinical practice is challenging. Among the difficulties facing family physicians are the differences in guidelines for various diseases, time constraints, and patients' difficulty in performing self-care for several diseases at the same time^7,10^. Previous research has described the impact of multimorbidity on the health system, addressing the high use of health services^8,9^, in cross-sectional^11^ and short-term cohort studies^12^.

### Objective

In this study, we followed a cohort of persons from the time of onset of type 2 diabetes for a period of 11 years. We assessed the utilization of health services and mortality risk according to the classification of concordant and discordant comorbidity.

## Results

**Figure 1:**
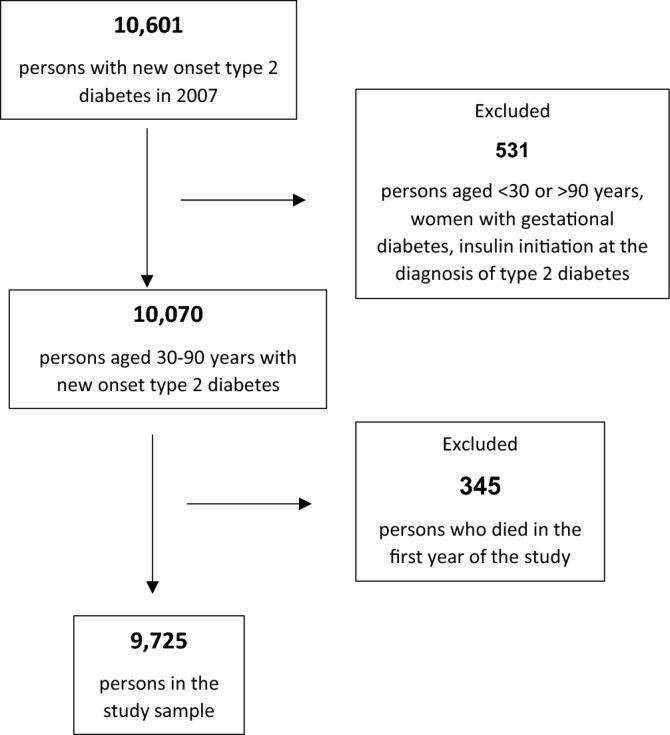
Flow chart of the study sample.

The study sample included 9725 persons who were identified during 2007 as having newly diagnosed type 2 diabetes according to the criteria delineated in the Methods (Fig. 1). The follow-up period lasted 9.7 ± 2.2 years/person, which contributed to the study’s 94,260 person years. After the first year, and until the end of the follow-up, 1785 (18%) persons died. Men comprised 52% of the cohort. The mean age at diabetes diagnosis was 58 ± 13 years, nearly half of the persons were aged 45–65 years and one third were aged 65 years and older (Table 1). The study sample comprised 54% Jews and 46% Arabs (Table 1). We identified 6.3 ± 3.8 diseases per person (Table 1). For 79% (n = 7209) of the persons in the cohort, comorbidities were detected both before and after diagnosis of type 2 diabetes. Lower rates were recorded for persons with comorbidities diagnosed only before (13%) or only after (8%) the diagnosis of type 2 diabetes.

**Table 1:** Demographic and morbidity characteristics of persons in various groups of comorbidities.

	All persons N (%)	Persons without comorbidities N (%)	Persons with concordant only comorbidities N (%)	Persons with discordant only comorbidities N (%)	Persons with combined comorbidity (concordant and discordant) N (%)	p value
**Gender**						
Men	5052 (52.0)	266 (48.4)	930 (57.5)	148 (60.9)	3708 (50.7)	< 0.0001
Women	4673 (48.0)	283 (51.5)	687 (42.5)	95 (39.1)	3608 (49.3)	
**Age at onset of diabetes**						
30–45	1529 (15.7)	176 (32.0)	426 (26.4)	106 (43.6)	821 (11.2)	< 0.0001
45–65	5165 (53.0)	201 (36.6)	1035 (64.0)	115 (47.3)	3814 (52.1)	
65	3031 (31.3)	172 (31.0)	156 (9.7)	22 (9.0)	2681 (36.6)	
**Ethnicity**						
Arabs	4451 (45.8)	105 (19.0)	937 (57.9)	74 (30.5)	3335 (45.6)	< 0.0001
Jews	5274 (54.2)	444 (81.0)	680 (42.0)	169 (69.5)	3981 (54.4)	
**Smoking**						
No	8892 (91.4)	510 (92.9)	1531 (94.7)	226 (93.0)	6625 (90.6)	< 0.0001
Yes (past and present)	833 (8.6)	39 (7.1)	86 (5.3)	17 (7.0)	691 (9.4)	
**Exemption from National Insurance payment**						
No	7897 (82.4)	459 (84.5)	1320 (82.5)	202 (84.5)	5916 (82.1)	0.4155
Yes	1688 (17.6)	84 (15.5)	279 (17.5)	37 (15.5)	1288 (17.9)	
**Death**						
No	7940 (81.7)	545 (99.3)	1548 (95.7)	215 (88.5)	5632 (77.0)	< 0.0001
Yes	1785 (18.3)	4 (0.7)	69 (4.3)	28 (11.5)	1684 (23.0)	
**Number of comorbidities (mean ± Std)**						
Total	6.3 ± 3.8	–	2.8 ± 1.6	2.0 ± 1.3	7.3 ± 3.6	< 0.0001
Before diabetes	3.8 ± 2.5	–	1.9 ± 1.1	1.5 ± 0.9	4.1 ± 2.5	< 0.0001
After diabetes	3.3 ± 2.4	–	1.9 ± 1.2	1.5 ± 0.8	3.6 ± 2.5	< 0.0001
**N total**	9725	549 (5.6)	1617 (16.6)	243 (2.5)	7316 (75)	

### Characteristics of persons with concordant and discordant comorbidities

**Figure 2:**
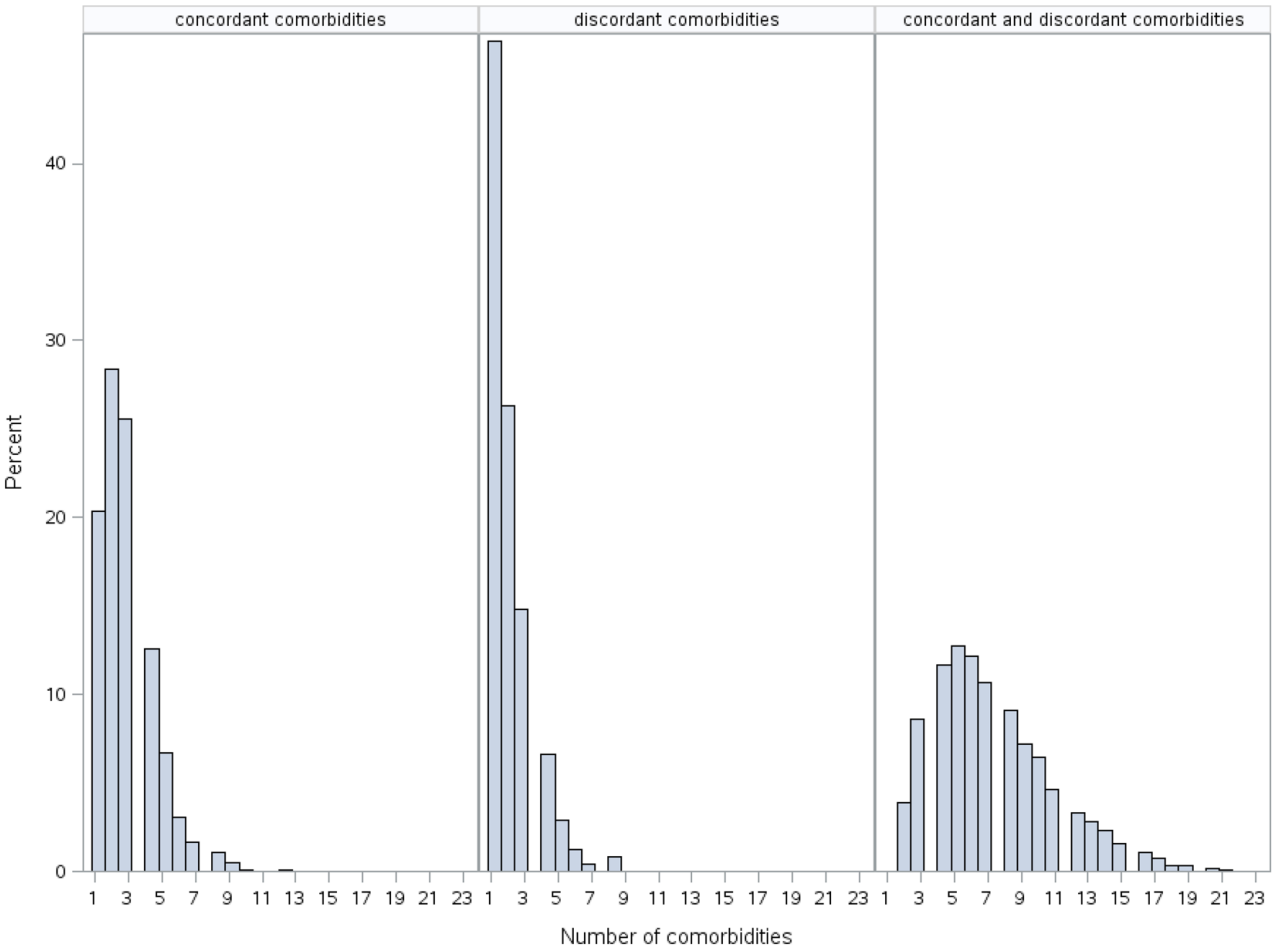
Distribution of number of diseases in persons from various groups of comorbidity. In persons with concordant or discordant comorbidities only, diseases were distributed across a smaller range compared to persons with combined comorbidities.

Diseases were grouped into concordant and discordant comorbidities according to the bodily systems affected (see Supplementary Tables S1 and S2 online). Persons with a combination of both concordant and discordant comorbidities, referred to here as “combined comorbidity”, were more prevalent (n = 7316; 75%) compared to persons with concordant only or discordant only comorbidities (16.6% and 2.5%, respectively). No comorbidity was reported in 549 (5.6%) persons. Men were more represented than women in all groups of comorbidities (Table 1). Persons aged 45–65 years at the diagnosis of type 2 diabetes were most highly represented in all comorbidity groups. Jews were dominant in the group of discordant comorbidities only (70%) and Arabs were dominant in the group of concordant comorbidities only (58%). Persons without an exemption from national health insurance payments were dominant in all groups of comorbidities (Table 1). The number of comorbidities per person was highest in the group of persons with combined comorbidities, compared to persons with concordant only or discordant only comorbidities (7.3 ± 3.6, 2.8 ± 1.6, 2.0 ± 1.3 respectively), with a gap remaining between the number of comorbidities before and after the onset of type 2 diabetes (Table 1). The distribution of the number of diseases between groups was different; in the groups of persons with concordant only or discordant only comorbidities, more persons had few diseases, while in the group of combined comorbidities, there was a broader range of diseases (Fig. 2).

A multinomial logistic regression model was designed to assess the risk of having any type of comorbidity of type 2 diabetes, considering the parameters of gender, age at onset of diabetes type 2, ethnic origin, smoking and exemption from national health insurance payments (Table 2). The reference group was persons without any comorbidity. Compared to persons aged 30–45 years at the onset of type 2 diabetes, those aged 45–65 years were at higher risk for having combined comorbidities [OR = 4.9; 95% CI 3.9–6.2), as were those aged 65 years or more [OR = 4.8; 95% CI 3.8–6.1]. Jews had a lower risk of having combined comorbidity compared to Arabs [OR = 0.2; 95% CI 0.2–0.3], similar to other groups of comorbidity. Women were at lower risk than men for having combined comorbidity [OR = 0.87; 95% CI 0.7–1.0], similar to other types of comorbidities. People with an exemption from social insurance payments had the same risk as those without it (Table 2).

**Table 2:** Multinomial logistic regression analysis for the risk of having concordant, discordant or combined comorbidities of type 2 diabetes compared to persons without comorbidities, according to various demographic characteristics.

Variables	Having concordant only comorbidities OR (95% CI)	Having discordant only comorbidities OR (95% CI)	Having combined comorbidities (concordant and discordant) OR (95% CI)
Gender (men)	1.00	1.00	1.00
Gender (women)	0.71 (0.58–0.87)	0.69 (0.51–0.95)	0.87 (0.72–1.04)
Age at onset of type 2 diabetes (30–45) years	1.00	1.00	1.00
Age at onset of type 2 diabetes (45–65) years	2.74 (2.15–3.49)	0.99 (0.71–1.40)	4.91 (3.92–6.15)
Age at onset of type 2 diabetes (65 +) years	0.61 (0.46–0.82)	0.24 (0.14–0.39)	4.84 (3.82–6.14)
Ethnic origin (Arabs)	1.00	1.00	1.00
Ethnic origin (Jews)	0.17 (0.14–0.22)	0.61 (0.43–0.87)	0.22 (0.18–0.28)
Smoking (no)	1.00	1.00	1.00
Smoking (yes–present and past)	0.77 (0.51–1.15)	0.97 (0.53–1.77)	1.37 (0.97–1.93)
Having no exemption from National Insurance payment	1.00	1.00	1.00
Having exemption from National Insurance payment	1.12 (0.85–1.46)	1.05 (0.69–1.60)	1.07 (0.84–1.37)

### Comorbidities and utilization of health services

‘Visits to family physicians’ was the most utilized health service during the study period, compared to visits to consultants and hospitalizations. Visits to family physicians were performed more by persons with combined comorbidities compared to persons with concordant only or discordant only comorbidities and persons without comorbidities (17.3 ± 10.2, 11.6 ± 6.5, 8.7 ± 6.8 and 6.3 ± 6.6 visits/person/year respectively, p < 0.0001) (see Supplementary Table S3 online).

**Figure 3:**
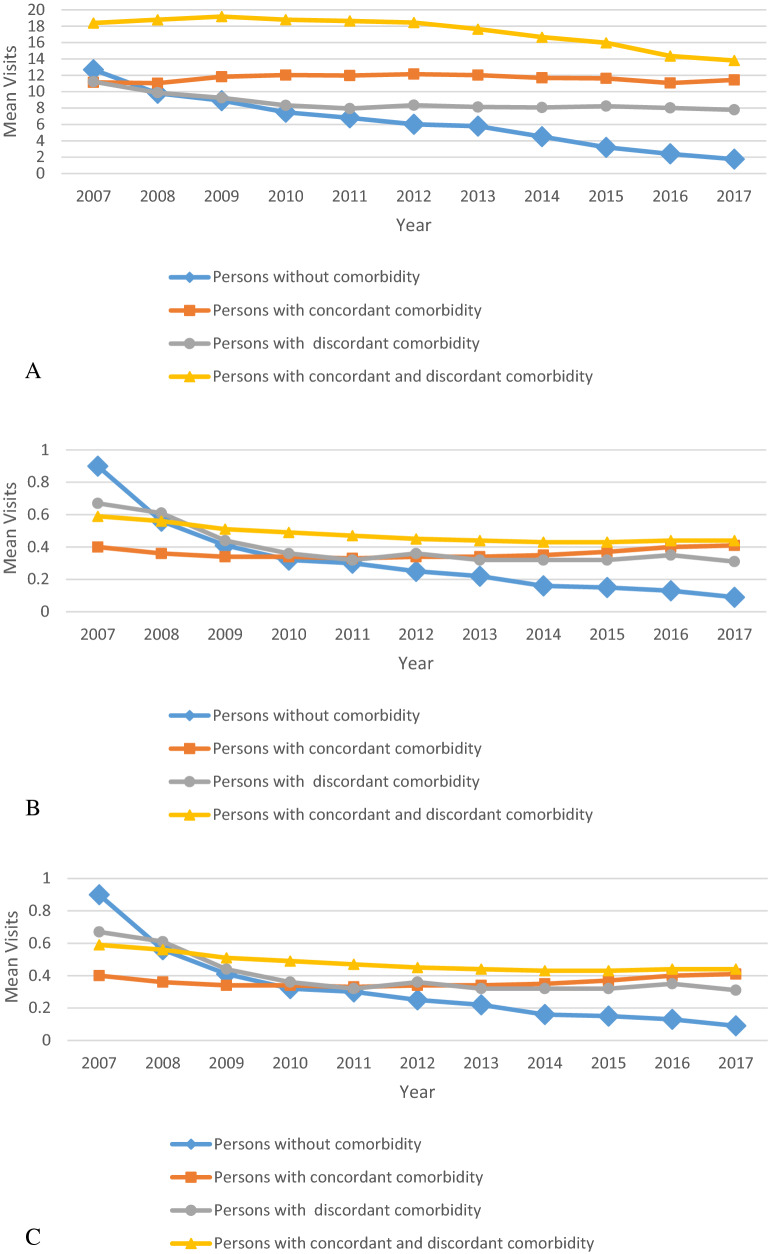
Annual health care services use visits to physicians in the community (mean visits/person/year or mean hospitalizations/person/year) and comorbidity during the study period. (**A**) Visits to family physicians; (**B**) visits to consultants; (**C**) hospitalizations (hospitalization/person/year).

The trend of annual use of health services presents differences between groups throughout the study period. Persons with combined comorbidity used them more than persons from other groups, and persons without comorbidity used them less than others; the differences between the groups were maintained over time (Figs. 3A–C). A downward trend in annual visits was observed among persons without comorbidities. Differences in hospitalization were steady throughout most of the study period with a tendency to rise in all groups, except in persons without comorbidity (Fig. 3C).

### Comorbidities and mortality

During the study period, 1785 (18%) persons died. The rate of death was highest among persons with combined comorbidity, followed by persons with discordant only comorbidities and concordant only comorbidities, and lowest among persons without comorbidity (23%, 11.5%, 4.3% and 0.7% respectively, p < 0.0001) (Table 1).

Incidence rates of death per 100 person years were 2.4 (95% CI 2.3–2.5), 1.2 (95% CI 0.7–1.6), 0.4 (95% CI 0.3–0.5) and 0.1 (95% CI 0.0–0.1), respectively.

Persons who died were more often older, Jews, smokers, and had more comorbidities than those who survived (see Supplementary Table 4 online). We performed a Cox regression analysis for risk of all-cause death, considering sex, age, ethnic origin, smoking and exemption from national health insurance payments. The reference group was persons who did not die. Persons with combined comorbidities and persons with discordant only comorbidities had a similarly higher risk of death [HR = 33.45; 95% CI 12.5–89.2 and HR = 33.48; 95% CI 11.7–95.8, respectively] than persons without comorbidities. Persons with concordant only comorbidities also had a higher risk of death [HR = 10.37; 95% CI 3.8–28.4], albeit lower than for persons with combined or discordant only comorbidities. Age 65 years and older at the onset of diabetes type 2 conferred a higher risk of death [HR = 14.7; 95% CI 10.75–20.22] than age 30–45 years. To a lesser extent, age 45–65 years also conferred a higher risk than age 30–45 years [HR = 3.2; 95% CI 2.3–4.4]. Women had a lower risk of death than men [HR = 0.8; 95% CI 0.7–0.9] (Table 3). No differences in risk of death were observed between Arabs and Jews, smokers and non-smokers, or between persons having or not having an exemption from national health insurance payments.

**Table 3:** Cox regression analysis for the risk of all-cause death in persons with type 2 diabetes, considering various types of comorbidities (compared to persons without comorbidities of type 2 diabetes) and sociodemographic characteristics.

Variable	Death (%)	HR	95% CI	p value
Having no comorbidities	4 (0.73)	1.00		
Having concordant only comorbidities	69 (4.27)	10.4	3.77–28.4	0.0001 >
Having discordant only comorbidities	28 (11.5)	33.5	11.7–95.8	0.0001 >
Having combined comorbidities (concordant and discordant)	1684 (23.0)	33.4	12.5–89.3	0.0001 >
Gender (men)	932 (18.4)	1.00		
Gender (women)	853 (18.2)	0.82	0.75–0.91	0.0001 >
Age at onset of type 2 diabetes (30–45) years	41 (2.68)	1.00		
Age at onset of type 2 diabetes (45–65) years	516 (9.99)	3.18	2.31–4.39	0.0001 >
Age at onset of type 2 diabetes (65+) years	1228 (40.5)	14.8	10.8–20.2	0.0001 >
Ethnic origin (Arabs)	689 (15.5)	1.00		
Ethnic origin (Jews)	1096 (20.8)	1.08	0.98–1.19	0.1075
Smoking (no)	1599 (18.0)	1.00		
Smoking (yes – present and past)	186 (22.3)	1.04	0.89–1.22	0.6188
Having no exemption from National Insurance payment	1420 (18.0)	1.00		
Having exemption from National Insurance payment	337 (20.0)	1.05	0.93–1.18	0.4276

The reference group was persons who did not die.

**Figure 4:**
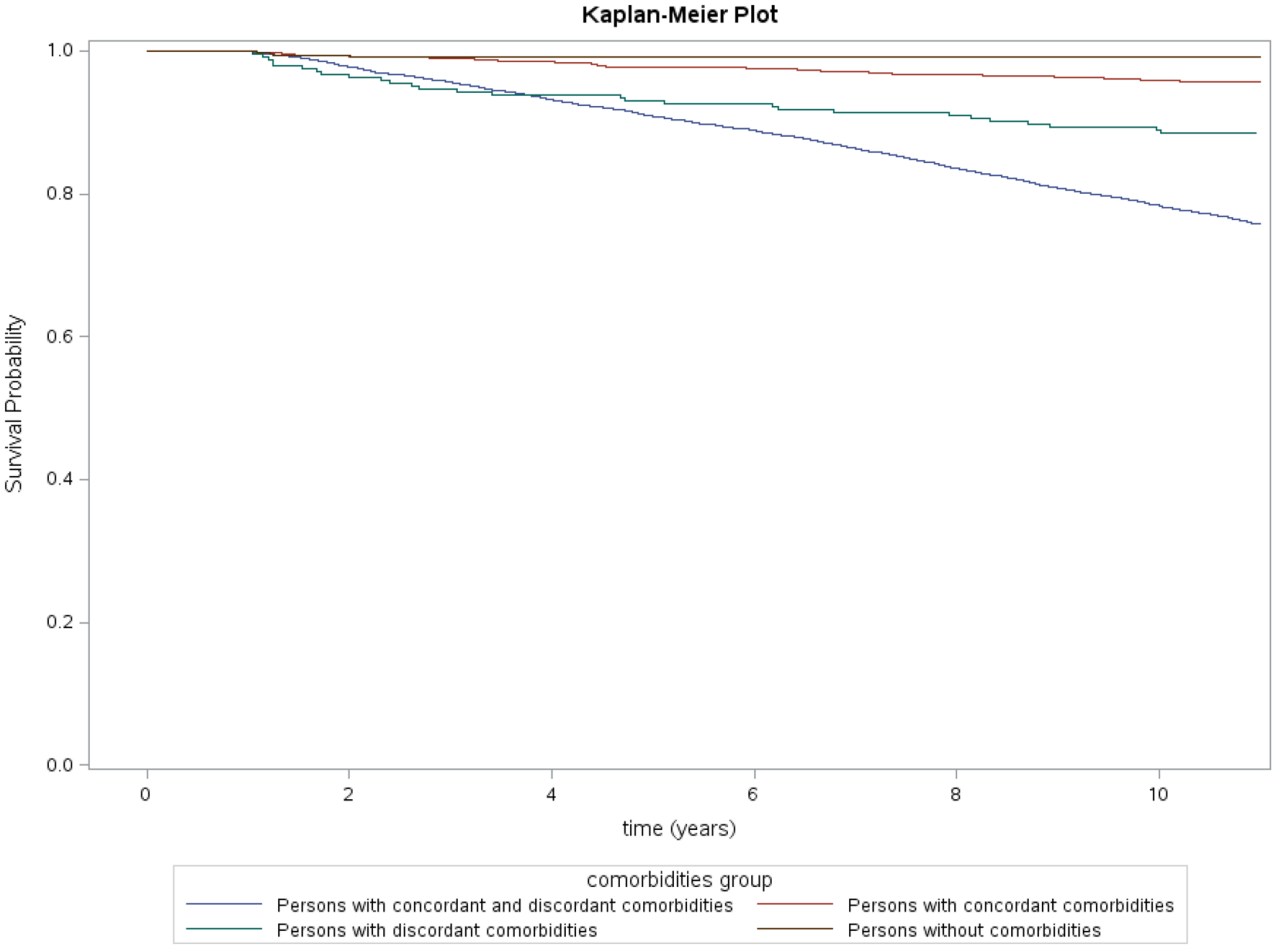
Kaplan–Meier plot of survival probabilities by comorbidity group: the comparison between comorbidities groups regarding time to death was significant using a Log-Rank test (p value < 0.0001).

We plotted the Kaplan–Meier curve to describe survival probabilities by comorbidity groups during the study period (Fig. 4). The steepest slope was observed in the group of persons with combined comorbidities, while the group of persons with discordant comorbidity only had a steeper slope than that of persons with concordant comorbidity only. The graph describing survival probabilities in the group of persons with no comorbidity was remarkably stable throughout the follow-up.

## Discussion

In this study of a large health maintenance organization, we followed persons from the time of onset of type 2 diabetes. At this time, most of the persons already exhibited other comorbidities. There were more individuals with both concordant and discordant comorbidities of type 2 diabetes than with either concordant or discordant comorbidities alone. The risk of all-cause death was highest in persons with combined comorbidity and in persons with discordant only comorbidities, indicating the contribution of discordant comorbidities to the outcome.

We defined the onset of type 2 diabetes as the starting point of our study, but we soon realized that the most common situation is that comorbidities, both concordant and discordant, appear before and after the onset of type 2 diabetes. The demonstration of prevalent comorbidities at the onset of diabetes corroborates previous reports^6,7,13^. This may be explained, at least in part, by the risk factors shared by type 2 diabetes and cardiovascular diseases, such as the metabolic syndrome^14^. The essence of type 2 diabetes may be a sequence of events in the development of comorbidities, rather than a definitive starting point.

The association of advanced age with comorbidities in persons with diabetes concurs with previous reports^3,4^. In our study, older age was associated with risk for both comorbidity and death.

Our study sample is characterized by near equal gender distribution; men had a higher risk for comorbidity and for death. A previous study reported higher prevalence in women than men of comorbidity in general, but not specifically of diabetes^3^.

Exemption from national health insurance payments, indicating low SES, was not associated with comorbidity and its outcomes, unlike previous findings^3,4^. This difference may be attributed to the national health insurance in Israel, which includes primary care services and hospitalization free of charge.

Arab ethnic origin was associated with higher risk for all categories of comorbidity. Considering cardiovascular diseases, which is the dominant concordance comorbidity, the outcome is in line with previous reports in Israel, of generally higher rates of cardiovascular diseases among Arabs than among Jews^15–17^. A novel aspect of the current study is the report of a higher rate of discordant comorbidity in the Arab population in Israel.

In our study, persons with combined comorbidities of type 2 diabetes consistently used health services more frequently than did persons with only concordant or discordant comorbidities. ‘Visits to family doctors’ was the health service most used in the community, more so among persons with combined comorbidity. The same trend was observed for hospitalization. Interestingly, among persons without comorbidities, the use of health services declined during the follow-up period. This could be due to better health or self-care. Our findings corroborate a report, based on private insurance company claims, of higher health service costs among persons with combined comorbidities than among persons with either discordant or concordant diseases alone^18^. In contrast to that study, our study is based on data from the national insurance health system. A recent publication from the Netherlands also reported higher societal costs of persons with diabetes and lower utilities^19^.

The death rate was higher in persons with combined comorbidities of type 2 diabetes, compared to only concordant or discordant comorbidities, or no comorbidities. The risk of death was similar for persons with combined comorbidities and discordant only comorbidities, although persons with discordant comorbidities only had fewer diseases. This raises the issue of the contribution of specific diseases, which is not addressed in our study. This may be consequent to the inclusion of malignant diseases in our study. Comorbidity of malignant diseases in persons with diabetes has been previously reported, and partly explained by shared risk factors^20,21^.

Other parameters that were identified as carrying higher risk for death in persons with type 2 diabetes were age 45 years and older, and male gender, in addition to comorbidities. Our study did not find Arab background to be an independent risk factor for mortality. Previous studies reported higher rates of all-cause mortality among Arabs, particular with respect to cardiovascular diseases^22,23^. However, combined comorbidities as defined herein were not reported in those studies.

We have not found another community-based study that compared mortality rates by concordance of comorbidities of type 2 diabetes. Previous publications that reported mortality in persons with diabetes focused on trends of mortality, showing that the decline in persons with diabetes lags behind those without diabetes^24–26^.

The strengths of our study include the long-term follow-up from the year of diagnosis of type 2 diabetes, the considerable sample size, and the real-life data of persons in the community. The large sample enabled us to draw conclusions regarding specific modes of the disease. This study is based on robust, valid sources of information, including the Clalit Health Services (CHS) chronic disease register, which is comprehensive and continuously updated^27^ with ongoing updated quality measures^28^.

Our study is limited by the crudeness of the data on type 2 diabetes, without accounting for specific diagnosis or severity of diseases, or for the individual level of control of type 2 diabetes or other chronic diseases. All-cause death was a study outcome, and not a specific cause of death. This is because the reason for death is not identified in the CHS registers. The analysis of death events using the Cox model was limited by the few cases recorded in the group of persons without comorbidities, which served as a reference group. As a result, the assumption of proportionality which underlies the Cox model was not met. Still, we consider it sensible to use the Cox model to examine the hazard as a function of the tested parameters during the study period.

Our data included services within the framework of public services. We do not have information on the use of private services, which could be relevant mainly for consultations. Our data did not include social information such as education or income. Instead, we used the exemption from national health insurance payments as an indicator of low SES. Since Israel operates a national health insurance system that provides services equally to all citizens, exemption from payment is an indirect indicator of SES. The health services assessed in our study are free of charge (visits to family physicians and hospitalization) or covered by a small copayment (visit to consultants). Medications are purchased at a subsidized price, which is adjusted for low SES and the number of medications. This health system thus narrows financial gaps for chronic patients. This could explain why SES was not found to be associated with the outcomes of our study.

In our databases, the validity of diagnosis of concordant diseases is high due to their inclusion in the Quality Measures program. Data related to discordant diseases may be less accurate, and could be under-reported. In our study, we grouped the chronic diseases in the list into systems. We tried to be most accurate in our grouping; however, some mis-grouping may have occurred. Malignant diseases were considered discordant diseases and may have contributed to the high rate of mortality. We chose not to exclude persons with malignant diseases, as in previous studies^9,12,29,30^, since this would necessitate excluding persons with other comorbidities as well. Furthermore, not all malignant diseases are terminal, and the presence of a malignant disease should not lead to lesser care for other comorbidities.

Discordant comorbidities have a significant effect on the outcomes of persons with type 2 diabetes both in combination with concordant comorbidities and without them. For further research, we suggest exploring the contribution of various discordant comorbidities, alone and in combination with concordant comorbidities, on the wellbeing and health of patients with type 2 diabetes. For persons with type 2 diabetes, this could lead to comprehensive recommendations for care that would consider both concordant and discordant comorbidities.

## Methods

### Design

This is a retrospective cohort study.

### Participants

The study included all adults aged 30–90 years on January 1, 2007, insured by CHS and residing within the northern district of Israel. Persons were included if they had a blood glucose test suggestive of new-onset type 2 diabetes in the year 2007, defined as a blood glucose level of ≥ 126 mg/dL (7.0 mmol/L) or a hemoglobin A1C of ≥ 6.5% (48 mmol/mol). Data were validated by the absence of a diagnosis of type 2 diabetes, or purchase of medications for type 2 diabetes during 2006. Study exclusion criteria were: gestational diabetes, insulin initiation at the diagnosis of type 2 diabetes and death recorded in the first year of the study. We excluded persons who died in the first year after inclusion so that the participants had at least one year of diabetes.

Israel operates a national health insurance scheme. Residents select one of four national health service providers, and an affiliated personal family physician^31^. CHS provides primary care to 70% of the inhabitants in the northern region of Israel. Primary care is provided in clinics by family physicians and consultants. Primary care clinics and health centers are dispersed throughout the captured area and accessible within a 30-min drive. Visits to family physicians are free of charge. Visits to consultants carry a low copayment. Admissions to hospitals are free of charge.

Persons were monitored until death or the end of the study period, December 31, 2017.

### Source of data

CHS operates an integrated electronic medical and administrative file, based on the International Classification of Diseases (9th Revision) combined with ICPC coding. All visits to doctors, either in the community or in hospitals, as well as hospitalizations, are recorded in the CHS database. Chronic conditions recorded in the CHS central register are based on reports by family physicians and community-based specialists, and hospital discharge letters. Chronic diseases that take part in the Quality Measures program, such as type 2 diabetes, cardiovascular diseases, asthma, and chronic obstructive pulmonary disease are also cross-validated against medication possession records and laboratory data through an automated disease-specific process^27,32^. We grouped diseases into bodily systems for analysis (Supplementary Table S1 online).

Dates of death (but not causes) are updated through direct linkage to the Israel Ministry of Internal Affairs, using a unique national personal identification number.

Baseline sociodemographic data included age at onset of diabetes, gender and ethnicity (Jewish or Arabs). The only available measure indicating life style behavior was smoking. The cohort was classified into three age groups: 30–45, 45–65 and 65 years and older. Eligibility for exemption from national health insurance payments is given to persons with very low income or who are unemployed for a long period, and was considered an indicator of low SES, as in previous studies^33,34^.

Type 2 diabetes and comorbidities were documented by the date of recording. We classified comorbid diseases using the system suggested by Piett and Kerr and developed by Mangan et al.^8,10^ (see Supplementary Table 2). Accordingly, concordant diseases share similar pathophysiology with type 2 diabetes and discordant diseases comprise the remaining pathologies. This classification was used in studies that evaluated quality of care, cost of care and self-care of type 2 diabetes^10,18,29,30^. Data on utilization of health services in the community, including visits to family physicians and consultants, were retrieved for all persons included in the cohort.

### Main measures

Persons were monitored for 11 years from the diagnosis of type 2 diabetes, stratified into groups according to whether comorbidities were concordant, discordant, combined (concordant and discordant) or absent. The use of health services and mortality were compared between the groups according to sociodemographic variables, the type of comorbidity and the number of comorbidities.

### Statistics

The data were analyzed using SAS version 9.4; p < 0.05 was considered significant. Categorical data were reported as numbers (%) and compared using the chi-square test. Multinomial logistic regression analysis was used to assess the risk of comorbidities of type 2 diabetes. A Cox regression model was used to identify factors that predict mortality. Continuous variables were reported as mean ± SD. Between-group comparisons regarding health services were performed using the Kruskal–Wallis test for non-parametric data.

Ethical approval was obtained from the CHS institutional ethics review board, with exemption from informed consent in data-based research using anonymous information (0137-17-COM2).

Statement: All methods were performed in accordance with the relevant guidelines.

## Supplementary Information

Supplementary Information 1.
