# Supplementary material for: Occurrence of comorbidities in newly diagnosed type 2 diabetes patients and their impact after 11 years’ follow-up
Source: Sci Rep. 2021 May 26;11:11071. doi: 10.1038/s41598-021-90379-0 (PMC8155151; doi:10.1038/s41598-021-90379-0)
Supplement: Supplementary file 1 — Supplementary Information 1. [file 41598_2021_90379_MOESM1_ESM.docx]

Occurrence of comorbidities in newly diagnosed type 2 diabetes patients and their impact after 11 years’ follow-up

Sophia Eilat-Tsanani, M.D.^1,2,3^

Avital Margalit, M.D.^1,2,3^

Liran Nevet Golan, M.D.^4^

1. The Department of Family Medicine,
2. Clalit Health Services, northern region
3. Azrieli Faculty of Medicine, Bar Ilan University,
4. Yoseftal Medical Center,

Supplementary Table S1. The list of chronic diseases categorized by systems

| **Disease** | **System** |
| --- | --- |
| **Acromegaly** | ENDOCRINOLOGY |
| **Addison’s disease** | ENDOCRINOLOGY |
| **Alcohol abuse** | ADDICTION |
| **Amputation of limb** | CARDIOVACULAR |
| **Amyloidosis** | IMMUNOLOGY/RHEUMATIC |
| **Anxiety** | PSYCHIATRY |
| **Aortic aneurism** | CARDIOVASCULAR |
| **Arrhythmia** | CARDIOVASCULAR |
| **Arthropathy** | IMMUNOLOGY/RHEUMATIC |
| **Asthma** | RESPIRATORY |
| **Behcet’s disease** | IMMUNOLOGY/RHEUMATIC |
| **Benign brain tumor** | MALIGNANCY |
| **Bipolar disease (manic depressive)** | PSYCHIATRY |
| **Blindness** | OPHTALMIC |
| **Bronchiectasis** | RESPIRATORY |
| **Congestive heart failure** | CARDIOVASCULAR |
| **Chronic obstructive pulmonary disease** | RESPIRATORY |
| **Cardiomyopathy** | CARDIOVASCULAR |
| **Carotid artery disease** | CARDIOVASCULAR |
| **Celiac disease** | GASTROINTESTINAL |
| **Chronic act/per hepatitis** | GASTROINTESTINAL |
| **Chronic bronchitis** | RESPIRATORY |
| **Chronic renal failure** | RENAL |
| **Cirrhosis** | GASTROINTESTINAL |
| **Crohn’s disease** | GASTROINTESTINAL |
| **Cushing’s disease** | ENDOCRINOLOGY |
| **Deafness** | EAR NOSE AND THROAT |
| **Dementia/Alzheimers/OMS** | NEUROLOGY |
| **Depression** | PSYCHIATRIC |
| **Diabetes insipidus** | ENDOCRINOLOGY |
| **Dialysis** | RENAL |
| **Drug abuse** | ADDICTION |
| **Eating disorders** | PSYCHIATRY |
| **Epilepsy** | NEUROLOGY |
| **Familial Mediterranean fever** | IMMUNOLOGY/RHEUMATIC |
| **Glaucoma** | OPHTALMIC. NON-DIABETIC |
| **Gout** | IMMUNOLOGY/RHEUMATIC |
| **Hemophilia** | HEMATOLOGY |
| **Hepatitis B carrier** | GASTROINTESTINAL |
| **Hepatitis C carrier** | GASTROINTESTINAL |
| **Hyperlipidemia** | HYPERLIPIDEMIA |
| **Hyperprolactinemia** | ENDOCRINOLOGY |
| **Hypertension** | CARDIOVASCULAR |
| **Hyperthyroidism** | ENDOCRINOLOGY |
| **Hypo/Hyperparathyroidism** | ENDOCRINOLOGY |
| **Hypophysis adenoma** | ENDOCRINOLOGY |
| **Hypothyroidism** | ENDOCRINOLOGY |
| **Ischemic heart disease** | CARDIOVASCULAR |
| **IHSS** | CARDIOVASCULAR |
| **Idiopathic thrombocytopenic purpura** | HEMATOLOGY |
| **Infertility male/female** | ENDOCRINOLOGY |
| **Irritable bowel syndrome** | GASTROINTESTINAL |
| **Joint replacement** | ORTHOPEDIC |
| **Kidney transplant** | RENAL |
| **Malignancy** | MALIGNANCY |
| **Motor neuron disease** | NEUROLOGY |
| **Multiple sclerosis** | NEUROLOGY |
| **Muscular dystrophy** | NEUROLOGY |
| **Myasthenia gravis** | NEUROLOGY |
| **Neuroses** | PSYCHIATRY |
| **Obesity** | METABOLIC |
| **Osteoporosis** | ENDOCRINOLOGY |
| **Other endocrine and metabolic disease** | ENDOCRINOLOGY |
| **Other hematologic disease (excluding iron deficiency anemia)** | HEMATOLOGY |
| **Other liver disease** | GASTROINTESTINAL |
| **Other neurological disease** | NEUROLOGY |
| **Other rheumatic/autoimmune disease** | IMMUNOLOGY/RHEUMATIC |
| **Peripheral vascular disease** | CARDIOVASCULAR |
| **Parkinson’s disease** | NEUROLOGY |
| **Pemphigus vulgaris** | DERMATOLOGY |
| **Peptic ulcer** | GASTROINTESTINAL |
| **Pernicious anemia** | HEMATOLOGY |
| **Polymyalgia rheumatica** | IMMUNOLOGY/RHEUMATIC |
| **Prostatic hypertrophy** | URINARY |
| **Psoriasis** | DERMATOLOGY |
| **Psychoses** | PSYCHIATRY |
| **Pulmonary hypertension** | RESPIRATORY |
| **Reflux esophagitis/Gastritis/Duodenitis** | GASTROINTESTINAL |
| **Retinitis pigmentosa** | OPHTALMIC. NON-DIABETIC |
| **Retinopathy** | OPHTALMIC |
| **Rheumatoid arthritis** | IMMUNOLOGY/RHEUMATIC |
| **Systemic lupus erythematosus** | IMMUNOLOGY/RHEUMATIC |
| **Sarcoidosis** | RESPIRATORY |
| **Schizophrenia** | PSYCHIATRY |
| **Scleroderma** | IMMUNOLOGY/RHEUMATIC |
| **Ulcerative colitis** | GASTROINTESTINAL |
| **Valvular cardiac disease (excluding mitral valve prolapse)** | CARDIOVASCULAR.DISCORDANT |
| **Other kidney disease** | RENAL |
| **s/p cerebrovascular accident** | CERBROVASCULAR |
| **s/p head of femur fracture** | ORTHOPEDIC |
| **s/p pulmonary embolism** | CARDIOVASCULAR.DISCORDANT |

Supplementary Table S2 ― Systems affected by chronic diseases categorized into concordant and discordant comorbidities

| Concordance | System | Frequency | Percent |
| --- | --- | --- | --- |
| Concordant comorbidities | CARDIOVASCULAR | 13867 | 24 |
|  | HYPERLIPIDEMIA | 7898 | 14 |
|  | METABOLIC | 5262 | 9 |
|  | NEUROLOGY* | 3288 | 6 |
|  | OPHTHALMIC* | 1239 | 2 |
|  | RENAL* | 3439 | 6 |
| Discordant Comorbidities | ADDICTION | 227 | 0.3 |
|  | CARDIOVASCULAR.DISCORDANT** | 1260 | 2 |
|  | CONGENITAL | 176 | 0.3 |
|  | DERMATOLOGY | 177 | 0.3 |
|  | ENDOCRINOLOGY | 2534 | 4 |
|  | EAR NOSE AND THROAT | 653 | 1 |
|  | GASTROINTESTINAL | 3366 | 6 |
|  | HEMATOLOGY | 459 | 0.8 |
|  | IMMUNOLOGY/RHEUMATIC | 4368 | 7 |
|  | MALIGNANCY | 1816 | 3 |
|  | OPHTHALMIC NON-DIABETIC*** | 624 | 1 |
|  | ORTHOPEDIC | 777 | 1 |
|  | PSYCHIATRIC | 2504 | 4 |
|  | RESPIRATORY | 2275 | 4 |
|  | URINARY | 758 | 1 |

* Concordant neurologic, ophthalmic and renal diseases are related to diabetes, and correlated to diabetic neuropathy, retinopathy and nephropathy respectively

**Cardiovascular-discordant – diseases without an ischemic background, for example congenital heart disease
*** ophthalmic non-diabetic – diseases that are not related to diabetes, such as cataract

Supplementary Table S3. Utilization of health services and concordance of diseases measured by visits/person/year

|  | All persons N=9725 | Persons without comorbidities (1) N=549 | Persons with concordant comorbidities (2) N=1617 | Persons with combined concordant and dis-concordant comorbidities (4) N=7316 | Persons with discordant comorbidities (3) N=243 | p-value |
| --- | --- | --- | --- | --- | --- | --- |
|  | Mean± SD | | | | |  |
| Family physicians | 15.5±10.0 | 6.3±6.6 | 11.6±6.5 | 17.3±10.2 | 8.7±6.8 | <0.0001 |
| Consultants | 0.4±0.3 | 0.3±0.2 | 0.4±0.2 | 0.5±0.3 | 0.4±0.3 | <0.0001 |
| Hospitalization | 0.7±1.4 | 0.3±0.8 | 0.2±0.97 | 0.8±1.4 | 0.6±3.1 | <0.0001 |

* Post-hoc analysis:

Visits to family physicians: All comparisons were significant

Visits to consultants: All comparisons significant except: (2) vs. (3)

Hospitalizations: All comparisons significant except: (1) vs. (2) and (1) vs. (3) and (3) vs. (4)

Supplementary Table S4. All-cause death – comparison between persons who died and those who survived according to demographic and morbidity characteristics

|  |  | No Death  N (%) | Death  N (%) | p-value |
| --- | --- | --- | --- | --- |
| Gender | Men | 4120(51.9) | 932(52.2) | 0.8046 |
|  | Women | 3820(48.1) | 853(47.8) |  |
| Age  at onset of diabetes | 30-45 | 1488(18.7) | 41(2.3) | <0.0001 |
|  | 45-65 | 4649(58.6) | 516(28.9) |  |
|  | 65+ | 1803(22.7) | 1228(68.8) |  |
| Ethnicity | Arabs | 3762(47.4) | 689(38.6) | <0.0001 |
|  | Jews | 4178(52.6) | 1096(61.4) |  |
| Smoking | No | 7293(91.9) | 1599(89.6) | 0.0019 |
|  | Yes (past and present) | 647(8.1) | 186(10.4) |  |
| Exemption from national security payment | No | 6477(82.7) | 1420(80.8) | 0.0560 |
|  | Yes | 1351(17.3) | 337(19.2) |  |
| Number of comorbidities (mean ±sd) |  | 5.2 ±3.5 | 9.3 ±4.1 | <0.0001 |
| N Total | | 7,940 | 1,785 |  |
